# Treatment of Hepatitis C as Prevention: A Modeling Case Study in Vietnam

**DOI:** 10.1371/journal.pone.0034548

**Published:** 2012-04-12

**Authors:** Nicolas Durier, Chi Nguyen, Lisa J. White

**Affiliations:** 1 Independent Research, Bangkok, Thailand; 2 FHI360, Hanoi, Vietnam; 3 Nuffield Department of Clinical Medicine, Centre for Clinical Vaccinology and Tropical Medicine, University of Oxford, John Radcliffe Hospital, Oxford, United Kingdom; 4 Faculty of Tropical Medicine, Mahidol University, Bangkok, Thailand; University of Sydney, Australia

## Abstract

**Background:**

Treatment of hepatitis C (HCV) is very effective, achieving a cure in 50–90% of patients. Besides its own good for individuals, this most likely translates in reduced transmission, but this phenomenon has yet to be fully explored.

**Methods and Findings:**

In this mathematical modeling study done in the context of Vietnam, we estimated the public health benefit that HCV therapy for injecting drug users (IDUs) may achieve. Treatment coverage of 25, 50 and 75% of chronically HCV-infected IDUs (4 years into infection) is predicted to reduce the chronic HCV viremia prevalence respectively by 21, 37 and 50%, 11 years after full scale up to the intended coverage. At a constant 50% coverage level, earlier treatment, 3, 2, and 1 year into infection is predicted to reduce the chronic HCV viremia prevalence by 46, 60 and 85%. In these later 3 scenarios, for every 100 treatment courses provided, a total of respectively 50, 61 and 94 new infections could be averted. These benefits were projected in the context of current low coverage of methadone maintenance therapy and needles/syringes exchange programs, and these services expansion showed complementary preventive benefits to HCV therapy. The program treatment commitment associated with the various scenarios is deemed reasonable. Our model projections are robust under adjustment for uncertainty in the model parameter values.

**Conclusions:**

In this case study in Vietnam, we project that treatment of HCV for injecting drug users will have a preventative herd effect in addition to curing patients in need for therapy, achieving a substantial reduction in HCV transmission and prevalence.

## Introduction

It is estimated that 130–170 million people around the world are chronically infected with the Hepatitis C Virus (HCV) [1]. HCV therapy is very effective, with 50–90% (depending on the virus genotype and epidemiological context) of people receiving the currently recommended regimen achieving a Sustained Virological Response (SVR), considered a cure of the infection [2]–[4]. HCV treatment is still prohibitively expensive and considered complex, and like HIV therapy 10 years ago, it is routinely offered in rich settings but almost entirely inaccessible in resource-limited settings. We propose that lessons learned on the HIV front be applied to overcome barriers to accessing HCV therapy in developing countries. One recent observation with HIV is that therapy, besides its own good for patients, is a potent prevention method that markedly reduces transmission [5]. With HCV, early mathematical modeling has now shown that treatment may also reduce transmission and result in HCV prevalence reduction [6], [7]. The available data though only derive from western contexts. Here, mathematical modeling was used in a case study in Vietnam to estimate the preventive effect that HCV therapy may have in a developing country context. This study focuses on the injecting-drug-users (IDU) sub-population, who carries the highest HCV burden. Although we appreciate that caring for IDUs presents particular challenges, we emphasize that evidence supports that compliance with therapy can be adequate in drug users [8], that similar treatment success rates can be observed in drug users and non-drug users [9], and importantly, that HCV reinfection after successful treatment of IDUs may be low [10]–[12].

## Methods

### The model

**Figure 1:**
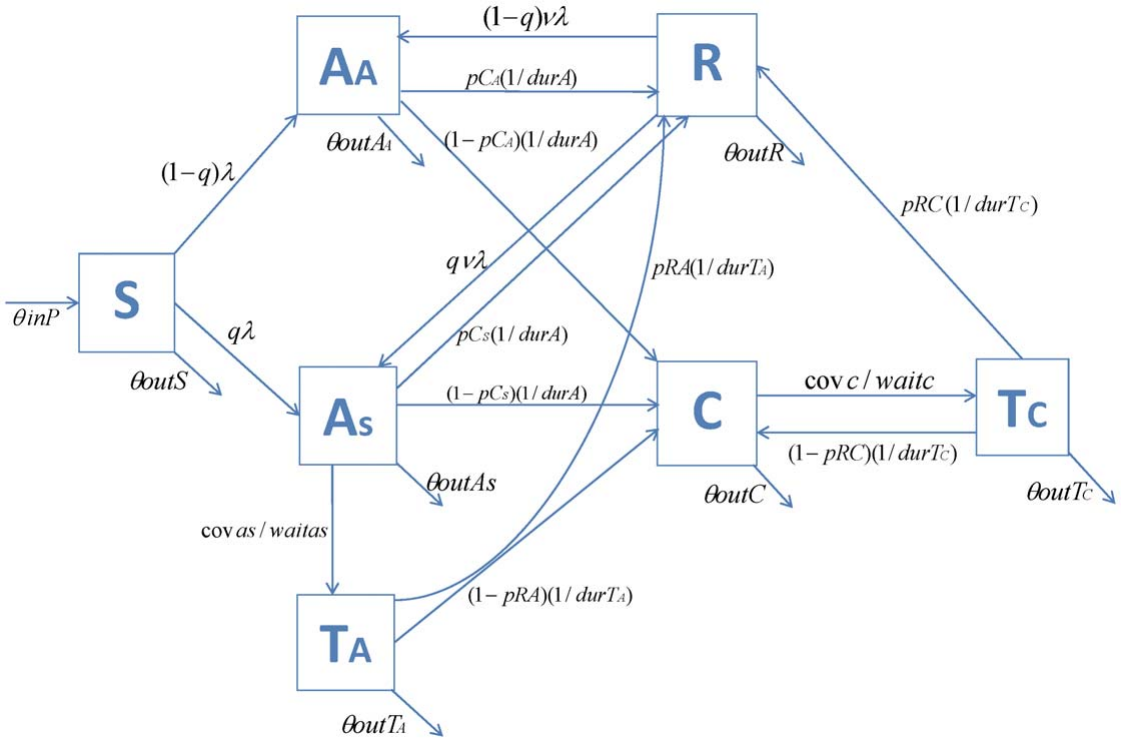
Model schematic. S: Susceptible individuals; A_A_: Acute asymptomatic cases; A_S_: Acute symptomatic cases; T_A_: Treated Acute symptomatic cases; R: Recovered infections; C: Chronic infections; T_C_: Treated Chronic infections.

The compartmental deterministic model, shown in Figure 1, considers the following groups of individuals: Susceptible (S) individuals who inject drugs but have not yet acquired HCV. Those who acquire infection are divided into acute asymptomatic (A_A_) and acute symptomatic cases (A_S_). Both subgroups may spontaneously clear infection/recover (R), or develop chronic hepatitis C infection (C). T_A_ and T_C_ represent respectively the acute symptomatic and chronic cases that will receive treatment. Both may either respond to treatment and recover from infection (R), or fail therapy and evolve/remain with chronic infection (C). Finally, cases that spontaneously cleared infection or were cured with treatment may be re-infected and re-enter the acute asymptomatic or symptomatic infection compartments.

The model includes the effect of Methadone Maintenance Therapy (MMT) and Needle-Syringe Exchange (NSE) programs. The IDUs are separated between those who respectively access, and do not access MMT, and movement takes place between the 2 sub-groups. In contrast, the NSE programs effect (*n_seeffect_*) are built into the model equations of force of infection. (Table 1)

**Table 1:** Model Equations.

Core Model Equations
*Individuals not on Methadone Maintenance Therapy*
*S_0_′ = θ_in_P−λ_0_S_0_−θ_out_S_0_−m_1_S_0_+m_2_S_M_*
*A_A0_′ = (1−q)λ_0_(S_0_+νR_0_)−((1/d_urA_)+θ_out_)A_A0_−m_1_A_A0_+m_2_A_AM_*
*A_S0_′ = qλ_0_(S_0_+νR_0_)−((1/d_urA_)+(c_ovAS_/w_aitAS_)+θ_out_)A_S0_−m_1_A_S0_+m_2_A_SM_*
*C_0_′ = (1−p_CA_)(1/d_urA_)A_A0_+(1−p_CS_)(1/d_urA_)A_S0_+(1−p_RA_)(1/d_urTA_)T_A0_+(1−p_RC_)(1/d_urTC_)T_C0_−((c_ovC_/w_aitC_)+θ_out_)C_0_−m_1_C_0_+m_2_C_M_*
*T_A0_′ = (c_ovAS_/w_aitAS_)A_S0_−((1/d_urTA_)+θ_out_)T_A0_−m_1_T_A0_+m_2_T_AM_*
*T_C0_′ = (c_ovC_/w_aitC_)C_0_−((1/d_urTC_)+θ_out_)T_C0_−m_1_T_C0_+m_2_T_CM_*
*R_0_′ = p_CS_(1/d_urA_)A_S0_+p_CA_(1/d_urA_)A_A0_+p_RC_(1/d_urTC_)T_C0_+p_RA_(1/d_urTA_)T_A0_−νλ_0_R_0_−θ_out_R_0_−m_1_R_0_+m_2_R_M_*
*And: λ_0_ = β(1−n_seeffect_)(A_A0_+A_S0_+C_0_+A_AM_+A_SM_+C_M_)/P*
*Individuals on Methadone Maintenance Therapy*
*S_M_′ = −λ_M_S_M_−θ_out_S_M_+m_1_S_0_−m_2_S_M_*
*A_AM_′ = (1−q)λ_M_(S_M_+νR_M_)−((1/d_urA_)+θ_out_)A_AM_+m_1_A_A0_−m_2_A_AM_*
*A_SM_′ = qλ_M_(S_M_+νR_M_)−((1/d_urA_)+(c_ovAS_/w_aitAS_)+θ_out_)A_SM_+m_1_A_S0_−m_2_A_SM_*
*C_M_′ = (1−p_CA_)(1/d_urA_)A_AM_+(1−p_CS_)(1/d_urA_)A_SM_+(1−p_RA_)(1/d_urTA_)T_AM_+(1−p_RC_)(1/d_urTC_)T_CM_−((c_ovC_/w_aitC_)+θ_out_)C_0_+m_1_C_0_−m_2_C_M_*
*T_AM_′ = (c_ovAS_/w_aitAS_)A_SM_−((1/d_urTA_)+θ_out_)T_AM_+m_1_T_A0_−m_2_T_AM_*
*T_CM_′ = (c_ovC_/w_aitC_)C_M_−((1/d_urTC_)+θ_out_)T_CM_+m_1_T_C0_−m_2_T_CM_*
*R_M_′ = p_CS_(1/d_urA_)A_SM_+p_CA_(1/d_urA_)A_AM_+p_RC_(1/d_urTC_)T_CM_+p_RA_(1/d_urTA_)T_AM_−νλ_M_R_M_−θ_out_R_M_+m_1_R_0_−m_2_R_M_*
*And: λ_M_ = αβ(1−n_seeffect_)(A_A0_+A_S0_+C_0_+A_AM_+A_SM_+C_M_)/P*

The intervention of key interest, HCV therapy, is only considered for acute symptomatic cases (T_A_, who have fair chances to present to or be reached by health services) and patients with chronic disease (T_C_). Acute asymptomatic cases are not considered for treatment due to the difficulty of reaching this group and the short duration of this state. Retreatment in those who failed therapy is not an option in this model (assuming a low probability that it would be offered in the programmatic conditions of interest), but it is possible for those re-infected after successful treatment, as they re-enter the pool of susceptible individuals. In a conservative manner, patients on treatment are considered infectious over the duration of therapy given that only a little over half achieve a complete Early Virological Response, i.e. an undetectable viral load by 12 weeks on treatment [13].

### Interventions scenarios

We studied 3 main intervention scenarios. In scenario A, we introduced HCV therapy at various coverage levels - 25, 50 and 75% - for individuals who have been infected for an average of 4 years (see parameters estimates). In scenario B, treatment coverage is fixed at 50%, but therapy is offered at increasingly early time points into chronic infection (4, 3, 2 and 1 year). It also then includes treatment of individuals with acute symptomatic infection at a 75% coverage level. Both scenarios are built in the context of current reach of MMT and NSE services, introduced in the model in 2007. In Vietnam, NSE services were introduced and expanded from 2005, and coverage has been roughly stable since 2007 [14]. MMT services appeared later with pilot projects started in 2008/9, and they have so far achieved very modest coverage [15]. The scenario C models an HCV therapy program covering 50% of individuals reached 2.5 years into chronic infection, in combination with expanded NSE and MMT services. In all scenarios, HCV therapy is introduced in 2012, but the selected coverage is only achieved after a scale up period of 4 years (see model equations), to mimic programmatic conditions. Likewise, expansion of NSE and MMT services in scenario C is modeled with a similar scale up period corresponding to national MMT services expansion plans [16].

### Outcomes

Conventionally, we first examined the effect of the interventions on the prevalence of hepatitis C infection. Although this logically included the prevalence of anti-HCV antibodies, we were here primarily concerned with the prevalence of true chronic viremic infections. Importantly, we also report the number of new infections averted per 100 treatment courses provided. For each scenario the model was run with and without treatment and the predicted cumulative number of cases and treatments were recorded. The number of cases averted was defined as the cumulative number of cases predicted from the model run without treatment minus the cumulative number of cases predicted from the model run with treatment. The number of cases averted per 100 treatment courses was defined as 100 multiplied by the cumulative number of cases averted and divided by the cumulative number of treatments predicted from the model run with treatment. The outcomes are examined up to only 2027, 15 years after treatment introduction and only 11 years after full scale up, to reflect a perspective directly appreciable by program and policy makers.

### Variables and Parameters Estimates

In Table 2, the variables/parameters point estimates derive from the best current available evidence, and are used to run the core simulations. When relevant, the ranges of parameter values are used to run sensitivity analysis (see sensitivity analysis).

*θ_in_* and *θ_out_* represent respectively the influx of new IDUs into the S compartment and the IDUs leaving any compartment as a result of death or injecting cessation. Although no published data could be found, experts in Vietnam estimate that the IDU population may have increased at an annual rate lower than 1% since 1990 (Quoc, Nguyen - FHI360, personnel communication). Therefore, *θ_in_* and *θ_out_* were set equal to maintain a constant population size in the core model, and varied in the sensitivity analysis. The *θ_out_* estimate corresponds to an injecting career length of 5.9 years, the average observed in IDUs in a large Integrated Biological and Behavioral Surveillance survey conducted in 10 provinces in Vietnam in 2009 (publication pending). Although some accounts, in other settings, have reported longer heroin use careers, some mixed non-injecting and injecting routes [17]–[19]. The Vietnam 2009 survey observed a total heroin use career of 7.9 years, and the apparent time lag between heroin initiation and injecting in this survey closely resembles that of another description [20]. *θ_in_* and *θ_out_* were also fitted simultaneously with *β* and *λ_0_* and *λ_M_* to reproduce the reported plateau HCV prevalence.
*β*, the transmission coefficient, was fitted to reproduce the HCV prevalence of 70–74% observed in IDUs in Vietnam between 2003–2006 [21], [22]. The resulting estimate infers a force of infection *λ_0_* = 0.43, which corresponds to a time between injecting onset to seroconversion of 2.3 years. It is believed to fit well the context. Indeed, although some studies from western settings have reported times from injecting onset to HCV infection of around 3.3 years [23], [24], a specific study in Vietnam oriented towards young injectors reported a time to seroconversion of 1.2 years [21].
*α*, is a reduced injecting rate coefficient among those on MMT, derived from findings in pilot MMT services where 21.6% on MMT tested positive for opioids [25]. We further consider that injecting in these individuals is reduced.
*m_1_* represents the baseline recruitment rate into the MMT program, and corresponds to 1.3% of IDUs being reached by this service in 2010 [15].
*m_1new_ is* the expanded recruitment into MMT, based on the Vietnamese MOH objective of reaching 53% IDUs by 2015 [16].
*m*_2_ is the drop-out from MMT observed in the pilot program in Hai Phong and Ho Chi Minh city [25].
*n_se_* represents the baseline NSE program achievements, and incorporates that in 2007 and 2009, with 50 needles distributed/IDU/year, only 10% of the IDUs' need for clean materials were covered in places where programs have been established [14].
*n_senew_* is the expanded NSE program achievement used in Scenario C, corresponding to an ambitious coverage of 100% of IDUs with 250 needles/IDU/year, a figure lower than the real estimated needs, but above a usual program target of 200 needles/person/year.
*d_urTA_* and *d_urTC_* represent the length of therapy for patients with respectively acute (24 weeks, conservative choice) and chronic HCV infection [26]. Although treatment duration varies in chronic infection for different genotypes (24–48 weeks), the figure used is also the conservative estimate for the genotypes case mix expected in the population of interest [4].
*p_RC_* is the overall sustained virological response (SVR) expected in the population of interest with chronic infection. It stems first from an estimated weighted average SVR (SVR = 0.78) expected for the case mix of prevalent genotypes in South-East and East Asia in well controlled settings [4]. It further considers a reduced efficacy fraction that would likely apply in programmatic conditions giving notably access to patients with HIV co-infection (30% of IDUs in Vietnam, IBBS 2009-publication pending). However, as recently reported in a meta-analysis [9], we estimated that SVR is not significantly different between IDUs (including active IDUs) and non-IDUs.
*p_RA_*, the treatment-induced SVR expected among patients with acute symptomatic infection, was also reduced from rates reported in more controlled conditions (SVR = 0.85) [27], [28].
*ν* is the reduced ratio of HCV acquisition expected in individuals who previously cleared infection (in response to therapy or spontaneously), as opposed to individuals without prior infection. It is derived from several small cohort studies which evidenced reinfection rates of 5% per annuum or less in IDUs successfully treated for HCV [10]–[12] and reduced chronic infection re-establishment following previous spontaneous clearance [29].
*w_aitC_*, the duration of infection before individuals with chronic HCV are treated, is based in Scenario A on an injecting career length when IDUs are reached and delay to seroconversion of respectively 6 and 2 years.

**Table 2:** Variables and Parameters Estimates.

Symbol	Parameter description	Estimate	Range in SA	Approach
*θ_in_*	Recruitment rate of susceptible IDUs	0.17/y	0.145–0.196	+/−15%
*θ_out_*	Exit rate (death and cessation)	0.17/y	0.145–0.196	+/−15%
*β*	Transmission coefficient	0.73	0.35–1.97*	
*λ_0_*	Force of infection in IDUs not on MMT	0.43	0.11–1.52**	
*λ_M_*	Force of infection in IDUs on MMT	0.043	0.011–0.152**	
BL prev	Baseline HCV antibody prevalence	72	41–90*	[33]
*α*	Reduced injecting coefficient in IDUs on MMT	0.1	0.085–0.115	+/−15%
*m_1_*	Baseline rate of recruitment into the MMT program	0.003	0.0025–0.0035	+/−15%
*m_1new_*	Expanded rate of recruitment into the MMT program	0.123	N/A	
*m_2_*	Rate of drop-out from MMT	0.14/y	0.119–0.161	+/−15%
*n_se_*	Baseline proportion of IDUs covered with sufficient clean injecting materials	0.1	0.085–0.115	+/−15%
*n_senew_*	Expanded proportion of IDUs covered with sufficient clean injecting materials	0.5	N/A	
*q*	Proportion of acute symptomatic infections	0.20	0.15–0.25	[34], [35]
*d_urA_*	Duration of acute HCV infection	0.5 y	N/A	[34]
*p_CA_*	Proportion of spontaneous clearance in acute asymptomatic infections	0.18	0.13–0.24	[36]
*p_CS_*	Proportion of spontaneous clearance in acute symptomatic infections	0.31	0.26–0.36	[36]
*d_urTA_*	Treatment regimen duration for acute cases	24 wks	N/A	[26]
*d_urTC_*	Treatment regimen duration for chronic case	48 wks	24–72	[26]
*p_RC_*	Proportion of treated chronic cases that recover	0.65	0.55–0.73	[4]
*p_RA_*	Proportion of treated acute symptomatic cases that recover	0.75	N/A	[27], [28]
*ν*	Reduced re-infection ratio in people with prior virus clearance	0.05	0.006–0.19	[10]
*C_ovC_*	Treatment coverage for chronic cases	25, 50, 75%	N/A	
*C_ovAS_*	Treatment coverage for acute symptomatic cases	75%	N/A	
*w_aitC_*	Length of infection before therapy in chronically infected cases	1–4 y	.–8	[18], [19]
*w_aitAS_*	Length of infection before therapy in acutely infected cases	12 wks	N/A	
*t_T_*	Duration for full HCV therapy scale up	4 y	.–8	
*t_N_*	Duration for full MMT and NSE scale up	4 y	N/A	

* *β* range selected to obtain the published range of baseline HCV Ab prevalence.

** *λ_0_* and *λ_M_* vary with *β*.

### Sensitivity Analysis

In both intervention scenarios A and B, we reexamined the model outputs after applying the estimated lower and upper values of all relevant parameters (Table 2). When available, published evidence was used. Otherwise, programmatically driven or arbitrary (+/−15%) variations were applied. A univariate extreme value sensitivity analysis was perfomed.

## Results

### Increasing coverage

**Figure 2:**
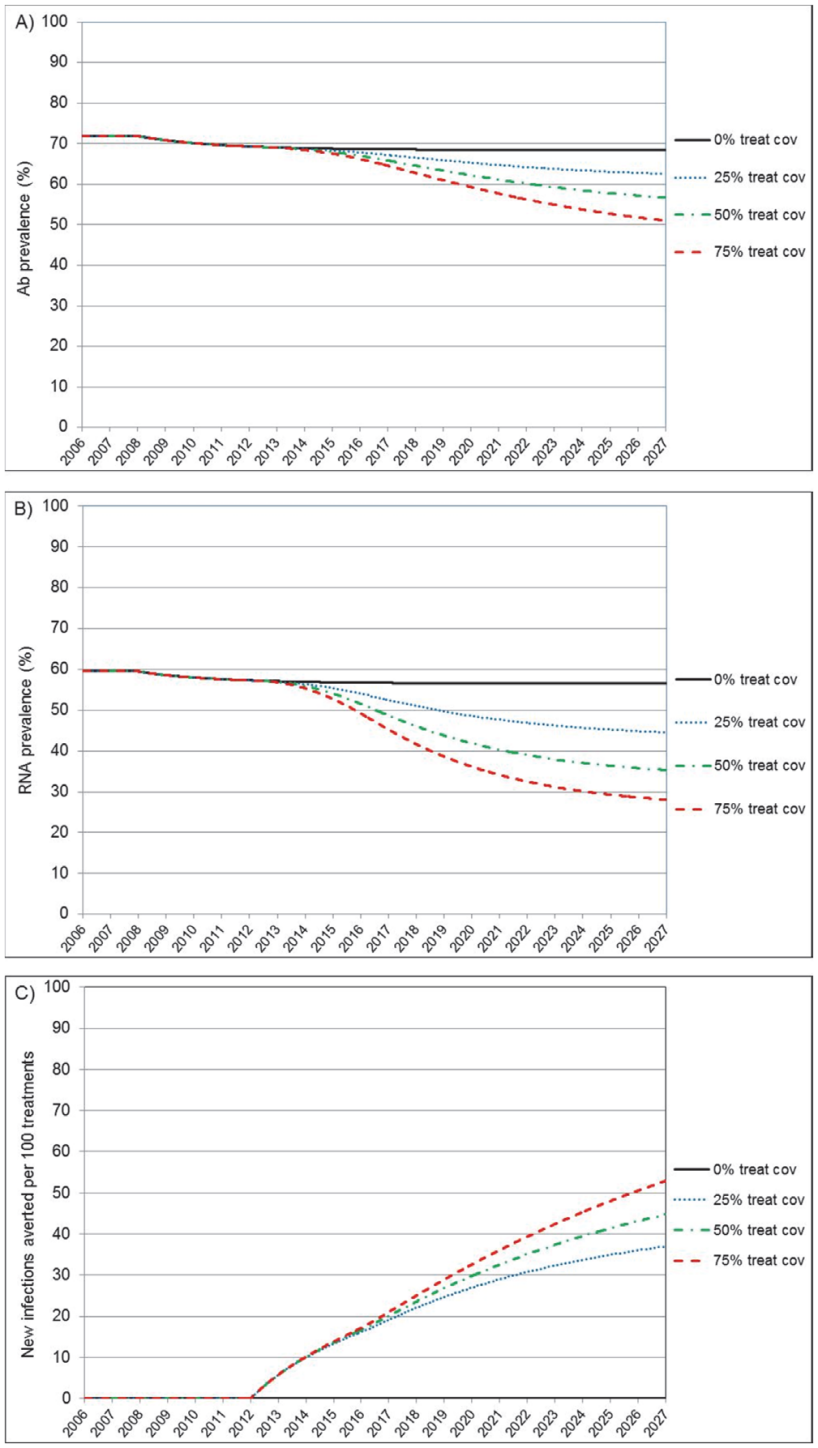
Projected preventive effect of increasing hepatitis C treatment coverage (Scenario A). Panel A): Reduction of anti-HCV antibody prevalence following treatment introduction and scale up to a 25%, 50% and 75% coverage level. Panel B): Reduction of prevalence of HCV true viremic chronic infections. Panel C): new infections averted per every 100 treatment courses of chronically infected cases.

Before interventions are introduced, the model accurately reproduced the HCV antibody endemic prevalence of around 72% reported in IDUs in Vietnam between 2003 and 2006. Modelling the introduction in 2007 of MMT and NSE programs current achievements predicted a very small prevalence reduction. (Fig. 2) No field observation is available to confirm this trend, but it is known that the HIV prevalence in IDUs has started to decline in Vietnam since 2005 and the start of harm reduction interventions [16]. Modelling the introduction and scale up of HCV therapy from 2012 predicts a very noticeable HCV prevalence reduction. The effect on the antibody prevalence appears smaller than that for chronic HCV viremia, as people cured from infection remain antibody positive. Increasing coverage levels predicted incremental benefits, such that by 2027 (11 years after full scale up), the 25, 50 and 75% coverage predict respectively a reduction of chronic HCV viremia prevalence of 21, 37 and 50%. Of importance, we project that at these respective coverage levels, every 100 treatment courses would prevent 37, 45 and 53 new infections, in addition to curing the 65% of patients offered treatment. This unique quantitative perspective has direct programmatic implications, especially with regard to cost-effectiveness.

### Treating earlier

**Figure 3:**
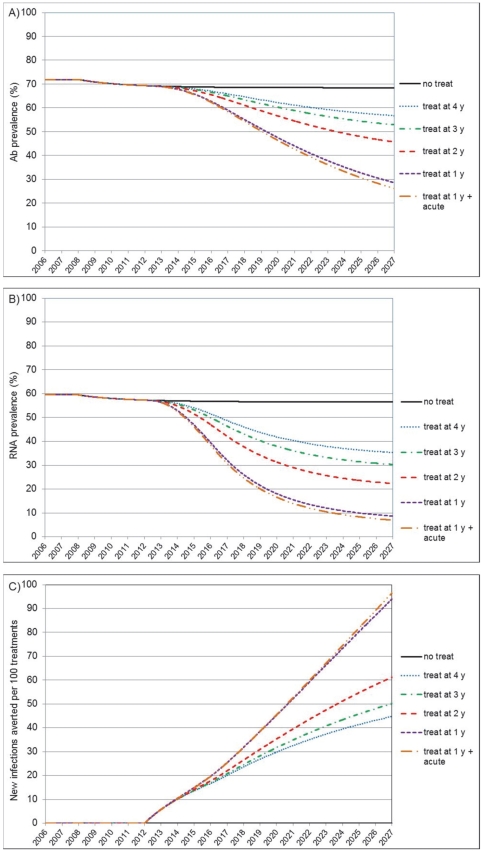
Projected preventive effect of treating earlier into infection (Scenario B). Panel A): Reduction of anti-HCV antibody prevalence following treatment of 50% of chronically infected cases, 4, 3, 2 and 1 year into infection, and 75% of acute symptomatic cases. Panel B): effect on chronic HCV viremia prevalence. Panel C): new infections averted per every 100 treatment courses initiated.

We further examined the impact of initiating treatment at an earlier point into patients' infection (Fig. 3). At a constant 50% coverage level, earlier treatment had remarkable incremental benefits. From the background 37% chronic viremia prevalence reduction seen if treating patients 4 years into infection (equivalent to scenario A), more proactive patient identification and recruitment into treatment 3, 2 and 1 years after established infection would result in a chronic viremia prevalence reduction of respectively 46, 60 and 85% after 11 years of fully scaled intervention. This illustrates the high number of transmitted infections that occur in every year of injection sharing. Eventually, the addition of an ambitious approach to identify and treat 75% of acutely infected symptomatic cases offers insignificant additional benefits, which is of no surprise considering the marginal time advantage, and the small proportion that acute symptomatic patients represent in the total case population. Predicted cases averted increase rapidly up to 94 averted infections per 100 treatments if treating patients 1 year into established infection. As an extension, we found that even at low coverage (25%), very early treatment (1 year) has important effects, reducing chronic viremia prevalence by 60% and averting 61 new infections per every 100 treatments (graph not shown).

### Combining treatment with expanded harm reduction programs

In Scenario C, starting HCV therapy to cover 50% of the IDUs 2.5 years into established disease offers a chronic HCV viremia reduction of 52%. The addition of expanded MMT services brings an additional prevalence reduction of 13% (down by 65%), and finally, expansion of NSE services adds a further 20% effect, bringing the chronic HCV viremia prevalence down to 9% after 11 years of full implementation (a total reduction effect of 85%). However, the addition of expanded MMT and NSE services does not greatly modify the number of infections averted per 100 treatment courses.

### Program commitment

We further projected that for every 1000 IDUs (our total model baseline population), a coverage of 75% of cases 4 years into infection (viremia prevalence reduction of 50%) corresponds to a cumulative number of 723 patients treated after 15 years of implementation. Intervening early (1 year) with 50% coverage (prevalence reduction of 85%) corresponds in turn to a cumulative treatment caseload of 947 patients after 15 years. On an average yearly basis, these 2 situations correspond respectively to 48 and 63 patients treated per year, for every 1000 IDUs. If extrapolating to the example situation of Hanoi (estimated IDU population in 2008 = 38,000 people [14]) these scenarios would correspond to treating respectively a total of 1824 and 2394 patients per year in the city.

### Sensitivity analysis

The sensitivity analyses showed our model predictions to be qualitatively robust. Effects observed were consistent in scenario A and B (Table 3, effects in scenario A only are presented). The outputs were virtually unaffected by the applied changes to *α*, *m_1_*, *m_2_*, *n_se_*, *q*, *p_CA_*, *p_CS_ and d_urTC_*. A doubling of the intervention scale up duration (*t_T_*) only marginally reduced benefits and the applied reduced rate in treatment response (*p_RC_*) had also little effect. As the applied lower and upper range of *p_RC_* correspond in turn to the SVR that may be expected in programmatic conditions for a subpopulation of patients infected with respectively genotype 1 or genotype 2–3, we project that the preventive effect of treatment may not be greatly modified in a program that would selectively target patients with more favorable genotypes. Importantly, the high estimate rate of reinfection (*ν*), corresponding to 100% re-infection after a little over 5 years, showed also very discrete reduction of the preventive benefits, as our model allowed treatment in case of reinfection. Outputs were more sensitive to variations in recruitment and exit rates, but a population increase with a higher influx of IDUs (which may more likely be happening in Vietnam according to experts) showed a noticeable increase in the projected prevalence reduction. Interestingly though, the later and new infections averted per 100 treatments changed in opposite direction with variation of *θ_in_* and *θ_out_*, as for example, a population increase would “dilute” prevalent cases and reduce the relative contribution of treatment on averted infections. Also, changes in the baseline HCV Ab prevalence influenced the model outputs, showing higher treatment preventive benefits at lower endemic levels, and yet a remaining 34% drop in chronic viremia prevalence in the extreme situation of a 90% baseline Ab prevalence. Finally, a doubling of the estimated time into infection before treatment (scenario A only) showed a noticeable reduction of treatment effect, yet with a persisting reduction of chronic viremia prevalence of 30%.

**Table 3:** Model outputs changes in sensitivity analyses.

	Scenario A (75% coverage, 4 years into infection)			
Parameter	Chronic viremia prevalence reduction	New infections averted/100 treatments	Chronic viremia prevalence reduction	New infections averted/100 treatments
Reference effect	50%	53	50%	53
Parameter	Low Estimate		High Estimate	
*θ_in_*	41%	62	60%	45
*θ_out_*	63%	36	40%	74
BL Ab prev	68%	96	34%	11
*α*, *m_1_*, *m_2_*, *n_se_*	50%	53	50%	53
*Q*	50%	52	50%	54
*p_CA_*	49%	48	52%	60
*p_CS_*	50%	52	50%	54
*d_urTC_*	52%	49	49%	56
*p_RC_*	45%	44	54%	60
*ν*	51%	56	47%	43
*w_aitC_*	N/A	N/A	30%	41
*t_T_*	N/A	N/A	48%	47

## Discussion

### Main Findings

In this study, we confirm previous predictions [6], [7] that treatment of chronic hepatitis C in injecting drug users may substantially reduce transmission of the virus and reduce prevalence. To our knowledge, ours is the first application in a developing country context, Vietnam in this circumstance. We found that even at low coverage levels (25%), HCV therapy results in an appreciable reduction of the prevalence of chronic HCV infection, and that more ambitious treatment programs could achieve a chronic viremia prevalence reduction of up to 85% after only 15 years of initiation and 11 years of full scale up. We show in particular that pro-active efforts to detect and treat patients early into infection offer rapidly increasing prevention benefits, owing to the high number of transmitting events that occur each year of injection sharing. We demonstrated these effects in the context of low coverage with harm reduction interventions (MMT and NSE), as currently observed in Vietnam, and show that expansion of these services would offer additive preventive benefits to an HCV treatment program.

### Implications

Worldwide, 130–170 million individuals are chronically infected with HCV [1]. Effective therapy exists and yet, a minority of patients in need (most live in developing countries) can access therapy. Here, we stress that treatment is not only beneficial for individual patients, and show that it is a plausible prevention method. At present, harm reduction methods, with other benefits, are considered the only option for controlling the spread of HCV among injecting drug users. Yet, often, they are the object of great controversy, domestically and among certain donors, and their implementation is painfully slow [15]. We argue and have provided new evidence that treatment of hepatitis C as prevention must be appreciated as a potential new tool to control the spread of this disease. We add, in due consideration of the current cost barrier to increased treatment access, that the true cost-effectiveness of this intervention must factor in its preventive effect. We showed in one treatment scenario that for every 100 patients started on treatment (of whom we expect 65% would be cured), 94 new infections could be averted. In gross terms, this would mean that the cost per treatment and per person should be halved, as each treatment would be expected to prevent roughly one additional infection.

### Strengths and limitations

Our sensitivity analyses, which included the application of extreme values to some of the parameters, showed our model outputs to be robust, and that the predicted HCV therapy preventive effects could apply in a range of different contexts, including within a wide range of baseline HCV prevalence, despite high reinfection rates, or reduced treatment efficacy. We have mimicked programmatic realities by building in a gradual scale up of the interventions of interest, rather than considering their introduction at a given instant as other modeling studies have done. We also projected and discussed the effects of the interventions at a perspective of 15 years post initiation and 11 years post full scale up, which may be of greater relevance to policy makers than a longer time horizon which would lead to predictions of greater effects. The following limitations are noted. Although we did not consider very unrealistic targets, such as elimination, we appreciate that some of our scenarios represent ambitious objectives. Many are skeptical of the feasibility of treating HCV-infected IDUs with ongoing substance use, despite published evidence that active IDUs can indeed be treated successfully [8], [9]. We recognize that successful programs require particular efforts and multidisciplinary interventions. Such care at a coverage level of 50% may prove very challenging, in particular in resource-limited settings. In turn, innovative interventions at the community level and using peers have helped filling some support gaps that may be unavailable in the institutional health sector [30]. In addition, we showed that even high treatment coverage in our models correspond to a manageable treatment caseload (financial considerations apart given the current treatment cost). Finally, we highlight that at low coverage level, although treatment would not greatly reduce prevalence, it would avert a substantial number of infections for every 100 treatment courses offered. Another limitation of our study is that it considered the integration of HCV therapy, MMT and NSE at the population level, rather than at the point of individual care. That is we do not model the mechanism of an HCV treatment program offering MMT and sufficient clean injecting materials to all treated subjects. However, the reality of services integration, as it is known with other compartments of care such as this of HIV and TB care, is complex. The implementation of MMT and NSE services is again often a sensitive matter, calling upon complex inter-sectorial engagement. As a result, we considered that the integration of HCV therapy, MMT and NSE services would realistically rely on referral between programs, and that complementarity would best be examined at the population level. As a next limitation, we did not consider the effect that changing prevalence of HIV or Hepatitis B co-infection could have on the risks of HCV acquisition or transmission. While our model was fitted to reproduce the HCV prevalence observed in IDUs at a time of 30% HIV co-infection, it is possible that this level of co-infection will vary over the coming 15 years, with a resulting effect that is not factored into this analysis. Then, although it is discussed that “susceptible” IDUs with recent onset on injecting have a different risk of HCV acquisition than more “experienced” injectors, we did not distinguish the 2 groups, as their distinct risks have not yet been well quantified [31]. Finally, we left out the risk of sexual transmission of HCV from IDUs, and only appreciate that we ignored a small (uncertain) number of infections averted to other groups. Acknowledging these limitations, we remain confident that they would not significantly alter the effects described in this study.

### Evidence from other studies

After projecting preventive benefits of HCV therapy in IDUs in the United Kingdom [6], Martin et al. used the optimal control theory to determine what the optimal HCV treatment programme strategy would be under a variety of policy objectives and budget constraints. They projected that an immediate programme of maximum intensity designed to minimize prevalence, HCV health utility loss and health services costs (which we extrapolate most closely matches our approach) would achieve greater cost-effectiveness, as opposed to programmes with notably delayed implementation addressing more restricted policy objectives or finite prevalence reduction targets [32]. In their study of optimal treatment allocation, Zeiler et al. determined that, as far as maximizing prevention benefits is concerned, HCV treatment should predominantly be allocated to IDUs not enrolled in MMT, as opposed to IDUs in MMT [7]. We stress that the concept of treatment allocation to optimize prevention benefits raises of course ethical dilemmas. In the present study, we introduce the important concept of timing of treatment, and project that the potential preventive benefits of HCV therapy are optimized if gains are made to treat patients earlier into their disease, or that at equal coverage, greater prevention effects are achieved if treating patients earlier into the infection.

### Further work

It is now needed to confirm and quantify in real-life the preventive effects of HCV therapy projected in this and other mathematical modeling studies. Similarly, it will be important to further study the cost-effectiveness of HCV therapy in developing countries with due appreciation of its direct benefits to patients and its indirect benefits to the population.
